# Formation of Hydrophobic–Hydrophilic Associates in the N-Vinylpyrrolidone and Vinyl Propyl Ether Copolymer Aqueous Solutions

**DOI:** 10.3390/polym15173578

**Published:** 2023-08-29

**Authors:** Sherniyaz Kabdushev, Grigoriy Mun, Ibragim Suleimenov, Adilet Alikulov, Ramazan Shaikhutdinov, Eldar Kopishev

**Affiliations:** 1 National Engineering Academy of the Republic of Kazakhstan, Almaty 050010, Kazakhstan; sherniyaz.kabdushev.hw@gmail.com (S.K.); mungrig@yandex.ru (G.M.); esenych@yandex.ru (I.S.); alikulov.adilet@gmail.com (A.A.); 2 Department of Chemistry & Technology of Organic Materials, Faculty of Chemistry and Chemical Technology, Polymers and Natural Compounds, Al Farabi Kazakh National University, Almaty 050040, Kazakhstan; ramossha123@list.ru; 3 Department of Chemistry, Faculty of Natural Sciences, L.N. Gumilyov Eurasian National University, Astana 010000, Kazakhstan; 4 Department of General and Inorganic Chemistry, Faculty of Natural Sciences, Bukhara State University, Bukhara 705018, Uzbekistan

**Keywords:** polymer solutions, hydrophobic interactions, hydrogen bonds, macromolecular coils, hydrophobic–hydrophilic associates

## Abstract

Utilizing turbidimetry data, an examination is conducted on the behavior of solutions containing N-vinylpyrrolidone and vinyl propyl ether copolymer within a temperature range coinciding with the occurrence of a phase transition. The investigation reveals that within specific conditions prevailing in this domain, the emergence of entities denoted as hydrophobic–hydrophilic associates is conceivable. These entities are characterized by the presence of a relatively dense core, upheld by hydrophobic interplays, and they are proficient in effectively dispersing irradiation within the optical spectrum. Encircling this core is a hydrophilic periphery that impedes the formation of insoluble precipitates. The development of such associates transpires when hydrophobic interactions have attained a discernible prominence, although they remain inadequate to counteract the forces that drive the expansion of macromolecular coils. Under these circumstances, the energetically favored course of action entails the constitution of a core for the aforementioned associates, involving discrete segments from diverse macromolecules. Notably, the introduction of an additional constituent (ethanol) to the solution, which selectively mitigates hydrophobic interactions, serves to stabilize the hydrophobic–hydrophilic associations.

## 1. Introduction

The enduring fascination with stimulus-sensitive polymers, which experience phase alterations upon the modulation of one or more thermodynamic variables, has persisted over recent decades [1,2,3,4]. This engrossment stems largely from practical imperatives. Evidently, polymers responsive to stimuli, along with the gels derived from them, hold immense potential as a highly promising substrate for finely tuned drug delivery systems [3,5,6,7,8], alongside their prospective applications within the realm of biomedicine [9,10,11,12].

Stimulus-sensitive hydrogels, manifesting a capacity for dynamic phase transitions, represent a propitious platform for the advancement of extraction technologies, including the selective isolation of diverse low-molecular-weight substances from solution matrices [13,14]. Furthermore, the reversible character inherent in these phase transitions, characterized by gel collapse, facilitates cyclic enrichment or depletion of the solution [15,16,17].

In the current juncture, the rationale for conceiving programmable architectures that elicit specific responses contingent upon predefined combinations of thermodynamic variables, or their sequential temporal permutations, is eminently justified [18,19,20]. Salient trends in this direction are already perceptible. The realization of intricate architectures, serving analogous objectives (e.g., through the modification of hydrogels), may be achieved by strategies such as the formation of complexes between cross-linked polymer networks and linear polymers [21,22], harnessing the agency of nanoparticles [23,24,25], exploiting interactions with heavy metal salts [26,27,28,29,30], or capitalizing on remote interplays between anionic and cationic hydrogels [31,32], among others.

Furthermore, as expounded in Reference [33], phase transitions can exhibit a hierarchically staged character, thereby engendering supplementary prospects for governing the responsivity of a cross-linked polymer network, or its composite derivatives, to fluctuations in thermodynamic parameters. This even extends to the potential for basic programmability within systems of this ilk [33].

Such conclusions extend beyond the purview of solely cross-linked polymer networks, thereby prompting contemplation of the future prospect of devising molecular programming systems engineered to manifest predetermined responses contingent upon specific permutations of thermodynamic parameters or their temporal dynamics. 

In the realm of solution dynamics, intricate structures of considerable complexity can indeed emerge due to the pronounced interplay among macromolecules. A case in point is the genesis of hydrophilic interpolymer associates (HIAs) [34,35,36], which materialize through interactions between ionic and nonionic macromolecular species.

HIA represents an intermediary entity, exhibiting features akin to both the classical interpolymer complex and the cross-linked network. It exists in a state of dynamic equilibrium, which is characterized by a continuous rupture and reformation of bonds linking dissimilar macromolecules.

This study underscores that intricate entities stemming from macromolecular interactions can also manifest in solutions composed of a solitary macromolecular species. To illustrate, empirical substantiation is presented, demonstrating the formation of entities within the solution encompassing N-vinylpyrrolidone and vinyl propyl ether (NVP-VPE) copolymer. Analogous to HIAs, these entities can be classified as hydrophobic–hydrophilic associates (HHAs).

The emergence of HHAs is contingent upon the same interplays that underlie the manifestation of phase transitions in temperature-responsive polymers, primarily of a hydrophobic nature, as temperature increases. Indeed, elevated temperature enhances hydrophobic interactions, culminating in phase transitions within solutions of macromolecules endowed with both hydrophilic and hydrophobic functional groups. Consequently, these transitions are intrinsically linked to a reconfiguration of the hydrophobic–hydrophilic equilibrium [35].

This recalibration precipitates a scenario wherein interactions among discrete macromolecular fragments begin to predominate over their interactions with solvent molecules, leading to a diminution in polymer solubility. This observable phenomenon is exemplified by the formation of precipitates.

Nonetheless, as demonstrated in this study, specific circumstances can arise wherein hydrophobic interactions attain a significant magnitude, yet their impact remains insufficient to yield a compact structural configuration within an individual macromolecule. In such instances, discrete segments from adjacent macromolecules congregate to form a condensed core stabilized by hydrophobic interactions. This core is enveloped by a periphery wherein interactions between macromolecules and the surrounding solvent hold sway. These are the very structures that warrant the appellation of HHAs.

Curiously, the significance of scrutinizing such systems, exemplified by HIAs and their analogous HHAs—whose existence is established in this research—extends well beyond the confines of polymer physical chemistry. Intriguingly, a bold hypothesis was advanced in [37], positing the universal ensemble as a neural network, or more precisely, its analogue. This conjecture harmoniously aligns with the findings of [38], which underscored the societal analogy to a neural network—a proposition further bolstered by mathematical models [39,40]. The outcomes delineated in [33], forming the foundation upon which polymer solutions may similarly be regarded as neural network analogues, complement the postulates set forth in [37,38,39,41]. Evidently, the inference can be drawn that intricate systems spanning a spectrum of diverse realms (in the philosophical sense of the term [42]) can be construed as akin to neural networks.

Undoubtedly, these deductions necessitate further substantiation, implying an imperative for the exploration of polymer solutions wherein entities akin to HIAs and their analog, HHAs, assume a role of genuine significance from a broader methodological vantage point. Furthermore, the parallel drawn with neural networks holds practical implications. In the contemporary literature, deliberations on the conception of computational frameworks founded upon intricate elemental substrates have gained prominence [43]. These discussions advocate for the utilization of quasi-biological constructs [43], nanotechnology-driven architectures [44], and related paradigms. Given the intimate interplay between cutting-edge computational systems and neural networks (pertinently exemplified by quantum computing systems [45,46] and optical neural networks [47,48]), an exploration of neural network attributes grounded in hydrophilic polymers emerges as an avenue of applied interest within this context.

## 2. Initial Experimental Data and Method of Their Processing

Among the array of techniques available for investigating phase transitions in solutions of stimulus-sensitive polymers, turbidimetry stands as a particularly potent tool. Through the acquisition of light-scattering intensity profiles contingent upon the manipulation of control parameters (such as temperature, pH, and the like), critical values of these parameters, signifying the junctures of phase transition, can be ascertained. This, in turn, furnishes insight into the mechanisms governing interactions within the solutions [49,50].

Nonetheless, it is not uncommon for such intensity profiles to exhibit monotonic behaviors, thereby engendering certain complexities in the precise quantitative determination of pivotal control parameter thresholds (e.g., the critical temperature for phase transition). As delineated in [51,52], a viable strategy to surmount this challenge emerges through the identification of a phenomenological regularity encapsulating the underlying dependencies. The work of [52] illustrates that under specific circumstances, the temperature and pH profiles governing turbidity within a solution of a stimulus-sensitive polymer align harmoniously with the subsequent equation. This equation, harnessed via the phase portrait methodology [53], offers a satisfactory depiction of the turbidity variations.

(1) $$Q=\frac{Q_{0}}{1+\exp\left(\left(T_{0}-T\right)/T_{q}\right)}+a$$

where $Q_{0}$ and a are the maximum and background values of the solution turbidity, respectively, $T_{q}$ is a parameter characterizing the width of the temperature range in which the phase transition occurs, and $T_{0}$ is the effective phase transition temperature. 

A similar expression finds applicability in representing turbidity dependencies with respect to pH [52].

It is noteworthy that the precise interpretation of the phase transition temperature within the context of monotonic dependencies remains an unresolved matter. In [51,52], a proposition was put forth to construe the phase transition temperature (or the critical pH value) as the temperature at which precisely half of the macromolecules in the solution undergo a change in their state. Given that Q≫a holds true, this criterion aligns with the value ${T=T}_{0}$, as directly deduced from Equation (1).

(2) $$Q\left(T_{0}\right)=\frac{Q_{0}}{2}+a\approx \frac{Q_{0}}{2}$$

It is pertinent to underscore that this Equation (2) is not intended for subsequent application; rather, its purpose lies in demonstrating that the peak of curve (1) indeed corresponds to the value of $\frac{Q_{0}}{2}$.

In the present study, this analytical approach is employed to analyze data previously garnered [54]. It merits emphasis that the advantage inherent to the technique expounded in [53] rests in its ability to pinpoint the phase transition temperature with a precision of 0.5 °C. It is this degree of accuracy that enables the discernment of effects linked to the emergence of HHAs.

## 3. Results and Discussion

**Figure 1:**
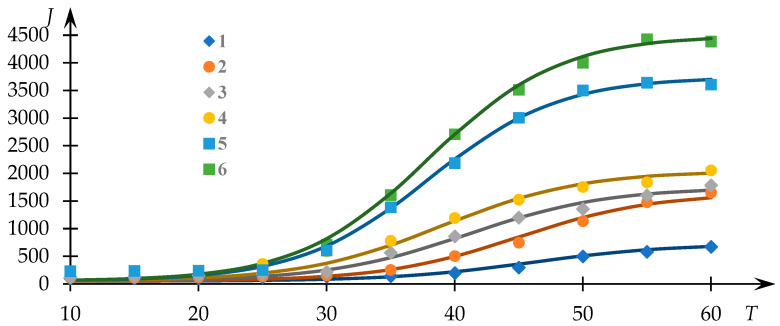
Light-scattering intensity dependencies (photocounts per second) against the temperature of NVP-VPE copolymer solutions at distinct concentrations; composition [NVP]:[VPE] = 74.5:25.5 mol%; c = 1 (1), 2 (2), 4 (3), 6 (4), 8 (5); 10 (6) mg/mL.

Figure 1 illustrates the experimental trends previously acquired [54], depicting the light-scattering intensity (measured in photocounts per second) as a function of temperature for solutions of the NVP-VPE copolymer at varying concentrations (indicated by data points). The continuous curves represent the fits generated via Equation (1), with the parameters $Q_{0}$, a, $T_{q}$ and $T_{0}$ determined through the least squares methodology.

**Figure 2:**
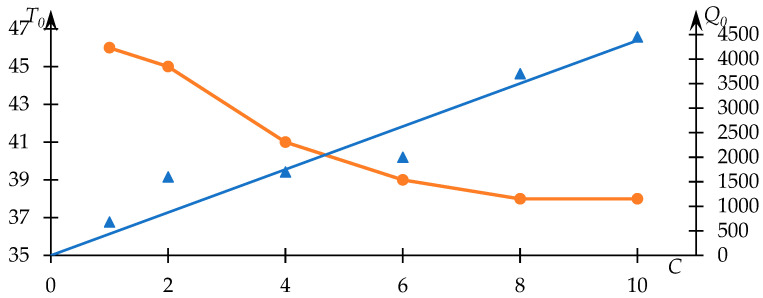
Dependence of the effective phase transition temperature $T_{0}$ of the NVP-VPE copolymer solution (blue line) and parameter $Q_{0}$ (orange line) on the copolymer concentration C in the solution.

Evidently, the approximation achieved through Equation (1) provides a satisfactory correspondence to the experimental dataset, thereby facilitating the extraction of phase transition temperature values and the parameter r $Q_{0}$ employing the approach elucidated in [53]. The variations of $T_{0}$ and $Q_{0}$ in relation to the concentration of the copolymer within the solution are presented in Figure 2.

Notably, the $Q_{0}\left(c\right)$ dependency displayed in Figure 2 showcases a predominantly linear tendency (passing through the origin, $R^{2}=0.97$, signifying a monotonic increment. Such a trend aligns with anticipation, as the light-scattering intensity subsequent to the phase transition completion (assuming absence of precipitate formation) ought to monotonically correlate with the polymer quantity within the solution. It was reasonable to anticipate a linear character for this relationship (a sufficiently close approximation to linearity is indeed observed in practice); however, a factor to consider is the occurrence of precipitate formation, which may introduce additional sources of error.

This outcome underscores the robustness of the employed data analysis method. Furthermore, the nature of the relationship between the phase transition temperature and copolymer concentration in the solution is in congruence with expectations. Higher concentrations correspond to more potent hydrophobic interactions, which are reflected notably in a decline of the phase transition temperature with increasing C.

**Figure 3:**
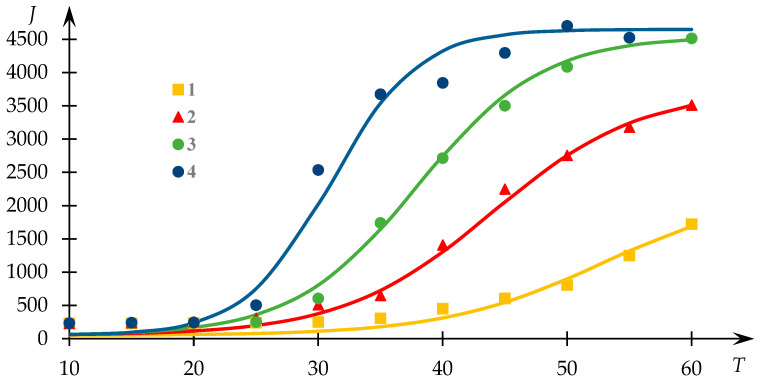
Light-scattering intensity dependencies J (photocounts per second) on temperature T for different NVP-VPE copolymer compositions; C = 10 mg/mL; composition x = [NVP]:[VPE] = 78.6:21.4 (1); 77.5:22.5 (2); 74.5:25.5 (3); 70.8:29.2 (4) mol%.

Dependence profiles of light-scattering intensity with respect to temperature for various compositions of NVP-VPE copolymer solutions are depicted in Figure 3. Experimental data points are sourced from [54], and the continuous lines depict the outcomes of the fitting process via Equation (1).

It is important to emphasize that although one of the curves in Figure 3 exhibits a dissimilar profile compared to the others, they nonetheless adhere to the same underlying regularity albeit different segments thereof. Specifically, Curve 2 in this illustration corresponds to cases where the transition to a plateau phase is successfully observed, whereas Curve 1 does not exhibit this characteristic. This divergence stems from the fact that the identification of the plateau phase in such curves is not always feasible due to the occurrence of precipitate formation, which may hinder its registration.

Evidently, the proposed approximation outlined in [34] aptly characterizes the experimental data in this instance.

**Figure 4:**
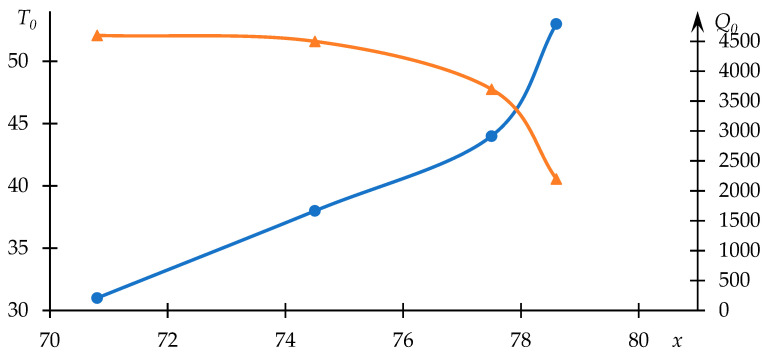
Variations of the effective phase transition temperature $T_{0}$ (blue line) of the NVP-VPE copolymer solution and parameter $Q_{0}$ (orange line) in relation to copolymer composition x.

The relationships between $T_{0}$ and $Q_{0}$ with respect to copolymer composition are depicted in Figure 4. As anticipated, the phase transition temperature decreases with an increase in the proportion of the component engaged in hydrophobic interactions.

Furthermore, it is evident that the dependency of parameter $Q_{0}$ on the variable parameter deviates from linearity—intensified hydrophobic interactions, leading to a notably rapid reduction in the phase transition temperature, are mirrored by heightened light scattering intensity.

**Figure 5:**
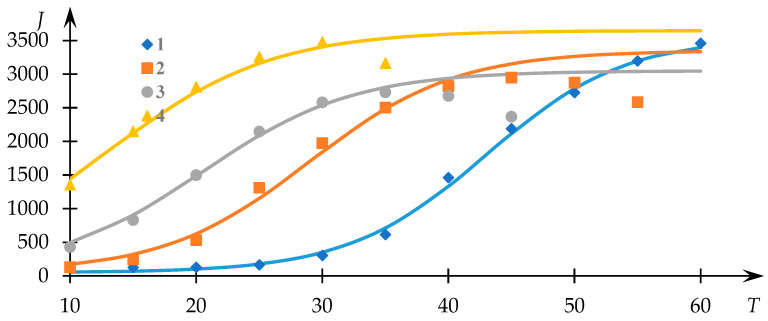
Light-scattering intensity dependencies J (photocounts per second) on temperature T of the NVP-VPE copolymer solution at distinct sodium chloride concentrations; composition [NVP]:[VPE]= 77.5:22.5 mol%; c = 10 mg/mL; $c_{NaCl}$ = 0 (1); 5 (2); 10 (3); 15 (4) wt. %.

**Figure 6:**
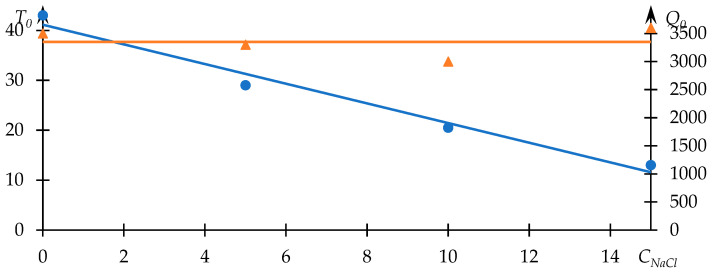
Trends in the effective phase transition temperature $T_{0}$ (blue line) of the NVP-VPE copolymer solution and parameter $Q_{0}$ (orange line) with respect to sodium chloride concentration $C_{NaCl}$, wt. % along with their linear approximations.

Comparable data, illustrating the influence of sodium chloride on the phase transition behavior, are displayed in Figure 5. Meanwhile, Figure 6 portrays the relationships between $T_{0}$ and $Q_{0}$ and the concentration of sodium chloride in the solution.

In this case, Equation (1) fittingly characterizes the experimental data. Furthermore, the observed alteration in the phase transition temperature aligns congruently with the existing literature. The presence of low molecular weight salt in the solution augments the manifestation of hydrophobic interactions. As the concentration of salt increases, the phase transition temperature demonstrates a monotonic decline, closely approximating a linear relationship (Figure 6).

In this instance, the $Q_{0}$ parameter exhibits a lack of conspicuous dependency on the concentration of salt. The parabolic approximation of the $Q_{0}$ concentration relationship, as depicted in Figure 6, remains closely aligned with a horizontal trajectory. This observation indicates that the turbidity of the solution following the occurrence of the phase transition remains virtually unswayed by alterations in salt concentration. This scenario corresponds to a circumstance wherein all macromolecules within the solution transition uniformly from one state to another. With the concentration of the polymer held constant, the number of macromolecules remains fixed. As a consequence, regardless of the notable influences exerted by additional factors, the turbidity of the solution subsequent to the completion of the macromolecular state change is expected to remain relatively constant. The form of approximation for the upper curve in Figure 6 may vary; specifically, a parabolic approximation is employed here with an $R^{2}=0.76$, although such a variation does not impact the formulated conclusion.

**Figure 7:**
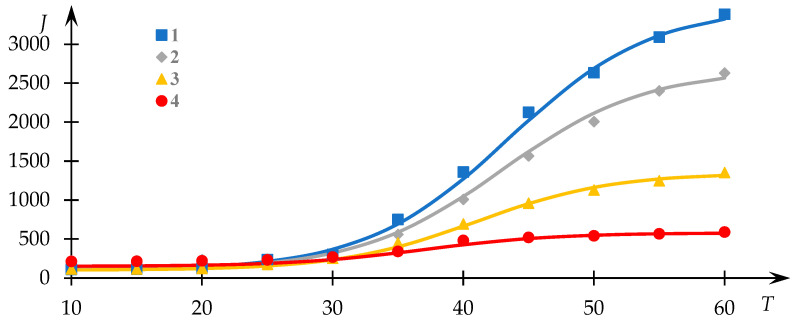
Intensity of light-scattering dependencies J (photocounts per second) as a function of temperature T for the NVP-VPE copolymer solution at various ethanol concentrations $c_{e}$; composition [NVP]:[VPE]= 77.5:22.5 mol%; c = 10 mg/mL; $c_{e}$ = 0 (1); 10 (2); 15 (3); 20 (4) wt. %.

**Figure 8:**
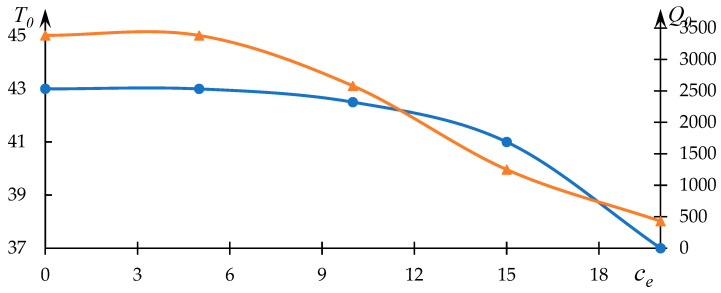
Variations in the effective phase transition temperature $T_{0}$ (blue line) of the phase transition of the NVP-VPE copolymer solution and parameter $Q_{0}$ (orange line) with ethanol concentration $c_{e}$.

The pivotal outcomes germane to the objectives of this study are depicted in Figure 7 and Figure 8. Resembling Figure 1 and Figure 3, Figure 7 encapsulates the impact of ethanol concentration on the characteristics of the phase transition. These depicted curves are similarly approximated by lines representing solutions to Equation (1).

The relationships between $T_{0}$ and $Q_{0}$ with respect to ethanol concentration $c_{e}$ are graphically depicted in Figure 8, and they are established upon the dataset in Figure 7.

Clearly observable in this instance is a noticeable decline in both the $Q_{0}$ parameter and the phase transition temperature as the ethanol concentration increases. This behavior is in accordance with the prevailing understanding that ethanol, to a certain extent, hinders the hydrophobic interactions entered into by the macromolecules.

At first glance, such behavior may appear paradoxical. Specifically, the substantial reduction in the $Q_{0}$ parameter with increasing ethanol concentration implies the inhibition of hydrophobic interactions. Normally, such inhibition would manifest in an elevation of the phase transition temperature, which is akin to the scenario elucidated earlier in relation to the influence of a low molecular weight electrolyte. However, actual observations reveal a contrary pattern. As will become evident from subsequent discussion, this incongruity points toward the formation of hydrophobic–hydrophilic associates.

Grounded in fundamental principles (affirmed, for instance, by mathematical models in [55]), the creation of any compact structure by one or more macromolecules necessitates the expenditure of effort against osmotic pressure, thereby countering the tendency for macromolecular coils to swell. Notably, a phase transition of the nature explored within this study is intrinsically tied to an adjustment in the hydrophobic–hydrophilic equilibrium consequent to temperature elevation.

Consequently, it becomes apparent that a specific range of conditions exists wherein hydrophobic interactions begin to emerge, yet it remains insufficient to provoke a conformational alteration in the swollen coils capable of counteracting osmotic pressure. Within this range, hydrophobic interactions between segments of distinct macromolecules are evidently poised to attain the utmost prominence.

**Figure 9:**
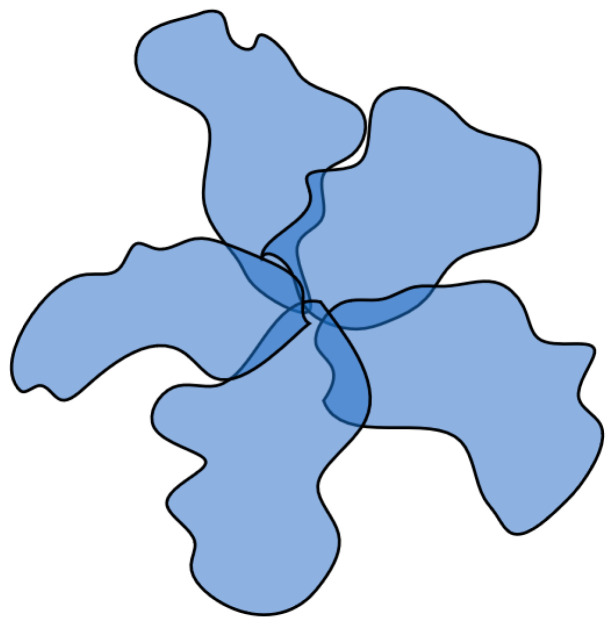
Schematic representation of a hydrophobic–hydrophilic associate structure formed by macromolecules of the same type.

The structure emerging under such circumstances parallels both the well-established “globule with edging” model and the conventional micelles formed by surfactants. Within this context, a relatively compact core takes shape, which is assembled from segments of multiple macromolecules facilitated by hydrophobic interactions. Concurrently, the extent of swelling in coils participating in the core formation remains notably elevated. This interplay gives rise to a supramolecular configuration, denoted as a hydrophobic–hydrophilic associate, which is characterized by a densely packed core capable of effective optical irradiation scattering and it is encompassed by a loosely bound periphery that maintains the structure’s solubility (Figure 9).

Hence, it can be contended that the phase transition induced by temperature elevation, at least within the context of the studied scenario, follows a staged progression. In the initial stage (at relatively lower temperatures), the aforementioned type of associates takes form. In the absence of factors impeding hydrophobic interactions, subsequent temperature increases prompt hydrophobic interactions between segments of the same macromolecule, resulting in diminished coil swelling within the core of the associate. Consequently, an increased number of macromolecules contribute to its assembly. This factor corresponds to the augmentation of associate cores, manifesting experimentally through heightened light-scattering intensity. At higher temperatures, the associate evolves into an insoluble particle as the hydrophilic periphery dissipates.

**Figure 10:**
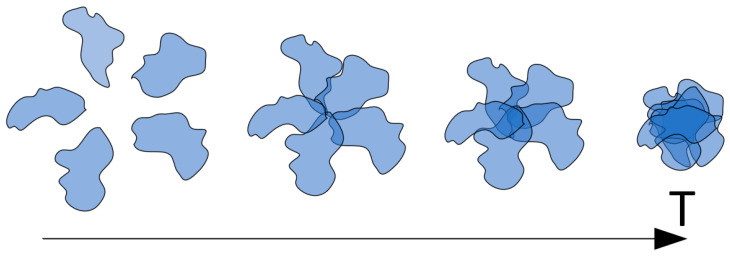
Schematic illustration of hydrophobic–hydrophilic associate transformations with increasing temperature.

The presence of factors partially obstructing hydrophobic interactions results in a scenario where the progression from non-interacting macromolecular coils to insoluble particles may be “frozen” at any of the stages (Figure 10), which is a phenomenon observed in experiments involving ethanol (Figure 7 and Figure 8).

In this context, the considered process halts at the stage of HHAs formation, with the density of the associate core contingent upon the degree of hydrophobic interaction blockage. Higher ethanol concentrations engender elevated coil swelling even at relatively elevated temperatures, thereby leading to lower core densities. Accordingly, the formation of relatively minor cores occurs across various ethanol concentrations. The stage schematically labeled as the first in Figure 10 transpires relatively smoothly, irrespective of the extent of hydrophobic interaction blockage, as the formation of small associate cores necessitates only a minimal alteration in initial coil swelling degrees. The stage identified as the second in Figure 10, associated with the formation of more substantial associate cores, may or may not transpire contingent upon ethanol concentration (or another agent obstructing hydrophobic interactions within the system).

In experimental observation, this manifests as a shift in the effective phase transition temperature toward lower values. It is noteworthy that this effective phase transition temperature corresponds to the inflection point within light-scattering intensity dependencies relative to the control parameter. As depicted in Figure 10, the emergence of HHAs featuring relatively small hydrophilic cores also transpires at relatively subdued temperatures. Consequently, if the subsequent progression of this process (i.e., the formation of HHAs with larger hydrophobic cores) is impeded, the effective phase transition temperature assumes a relatively modest value, reflecting solely the initial phase transition stage mentioned earlier. Conversely, should the process advance, leading to the formation of HHAs with substantial cores, the effective phase transition temperature will shift toward higher values. This alignment concurs with the gleaned experimental data (Figure 8).

The most disputable aspect of the conclusions articulated above pertains to the deduction of the existence of hydrophobic–hydrophilic associates. We emphasize that this concept is introduced for the first time in this work. To further substantiate the inference of their existence, we put forth a theoretical model, which is expounded upon in subsequent discourse.

## 4. Model for the Formation of Hydrophobic–Hydrophilic Associates

**Figure 11:**
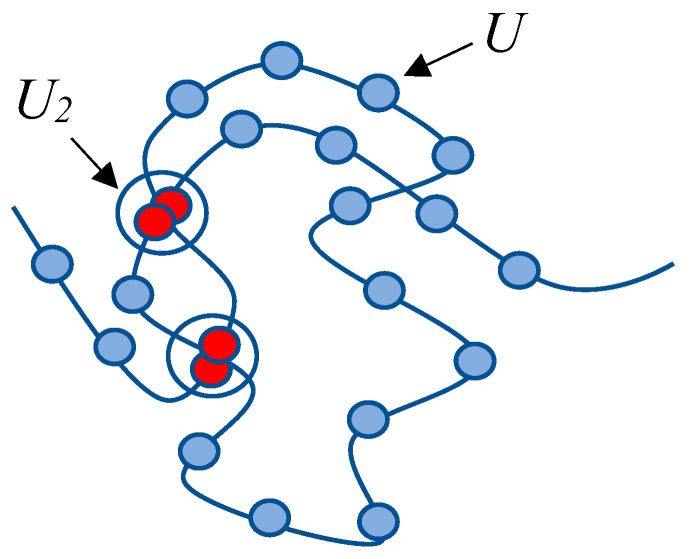
Simplified scheme illustrating the formation of a compact structure of macromolecular coils through hydrophobic interactions.

The illustrative model presented below aims to establish the existence of hydrophobic–hydrophilic associates, i.e., complexes formed by identical macromolecules within a solution (supramolecular entities formed by multiple identical macromolecules). It is important to emphasize that the proposed model is of an illustrative nature: it is intended to demonstrate the potential formation of hydrophobic–hydrophilic associates—not to determine their specific characteristics. For the simplification of calculations, we employ an exceedingly basic transition–coil–globule model based on the following idealization. Instead of considering a macromolecule with functional groups capable of engaging in hydrophobic interactions with both identical functional groups and those of neighboring macromolecules, we examine a collection of “beads on a string.” It is postulated that each node (each “bead”) can participate in a reversible chemical reaction with another similar node (Figure 11).

(3) $$U+U↔U_{2}$$

Within the framework of this highly simplified model, the sizes of the coil structures are determined by the swelling of subchains confined between interacting nodes, corresponding to the “product” of reaction (3).

The reversible nature of the formal reaction (3) permits the depiction of the balance between hydrophobic and hydrophilic interactions within the considered system. This balance is reflected in the shift of the formal reaction (3) to the left or right, which is contingent upon conditions governed by control parameters. The equilibrium in reaction (3) is characterized by the constant *K*, which is conventionally defined as

(4) $${\left[U\right]}^{2}=K\left[U_{2}\right]$$

It is worth underscoring that this model engenders a competition between internal hydrophobic interactions and those involving functional groups belonging to distinct macromolecules. This factor, as elucidated further, enables an analogy to be drawn between systems of the studied type and neural networks.

At the heart of the proposed model lies an integral describing the effect of two macromolecular coil clusters overlapping. Specifically, for the case of Gaussian coils, this integral can be precisely computed. The density of macromolecular segments $\rho \left(\vec{r}\right)$ within a Gaussian coil is described by the formula.

(5) $$\rho \left(\vec{r}\right)\sim \exp\left(-\frac{{\vec{r}}^{2}}{R_{0}^{2}}\right)$$

Let us define the function $\alpha_{0}\left(\left|{\vec{r}}_{0}\right|\right)$, characterizing the extent of overlap between two coils positioned at a distance $r_{0}$ from each other.

(6) $$\alpha_{0}\left(\left|{\vec{r}}_{0}\right|\right)=\alpha_{1}\int \exp\left(-\frac{{\vec{r}}^{2}}{R_{0}^{2}}\right)\exp\left(-\frac{{\left(\vec{r}-{\vec{r}}_{0}\right)}^{2}}{R_{0}^{2}}\right)dV$$

This same function determines the potential of segments belonging to two different macromolecules to interact with each other, as the integrand signifies the likelihood of such segments occupying the same region of space.

The normalization coefficient can be determined from the condition that when the coils are completely overlapped, α equals 1.

(7) $$\alpha_{1}\int \exp\left(-2\frac{{\vec{r}}^{2}}{R_{0}^{2}}\right)dV=1$$

The definite integral appearing in the right-hand segment of equation (6) is amenable to explicit evaluation, as the expression situated beneath the exponent is subjected to a full quadratic transformation:

(8) $$\frac{{\vec{r}}^{2}}{R_{0}^{2}}+\frac{{\left(\vec{r}-{\vec{r}}_{0}\right)}^{2}}{R_{0}^{2}}=2\frac{{\vec{r}}^{2}}{R_{0}^{2}}-2\frac{\vec{r}{\vec{r}}_{0}}{R_{0}^{2}}+\frac{{\vec{r}}_{0}^{2}}{R_{0}^{2}}=2\frac{{\left(\vec{r}-\frac{{\vec{r}}_{0}}{2}\right)}^{2}}{R_{0}^{2}}+\frac{{\vec{r}}_{0}^{2}}{2R_{0}^{2}}$$

By employing expression (8) and performing a change of variables in the integrand (permissible since the integral is evaluated over infinite limits), we derive

(9) $$\alpha_{0}=\alpha_{1}\exp\left(-\frac{{\vec{r}}_{0}^{2}}{{2R}_{0}^{2}}\right)\int \exp\left(-2\frac{{\vec{r}}^{2}}{R_{0}^{2}}\right)dV$$

Evidently, the normalization coefficient can be obviated from explicit computation. Precisely, it holds that $\alpha_{0}$ is given by the expression:

(10) $$\alpha_{0}=\exp\left(-\frac{r_{0}^{2}}{{2R}_{0}^{2}}\right)$$

However, a nuance exists. Formula (10) captures the interaction between only two macromolecular clusters. In reality, each given macromolecular cluster in a solution is surrounded by a sufficiently large number of neighbors. Their count is deduced from formal geometric considerations.

Each macromolecule corresponds to a volume in the solution equal to the volume of a sphere with a radius taken as $\frac{r_{0}}{2}$, i.e., half the distance between the centers of mass of the macromolecules.

(11) $$\frac{4\pi }{3}C{\left(\frac{r_{0}}{2}\right)}^{3}=1$$

where *C* represents the concentration of macromolecules in the solution (the number of macromolecular clusters per unit volume); $V_{m}=\frac{4\pi }{3}{\left(\frac{r_{0}}{2}\right)}^{3}$ is the volume of solution assigned to an individual macromolecular cluster. The number of neighboring macromolecules can be determined from the following considerations. These macromolecules account for the volume of a sphere with a radius of $\frac{3r_{0}}{2}$ minus the volume of a sphere corresponding to the central macromolecule, i.e.,

(12) $$n_{s}=\frac{4\pi }{3V_{m}}C\left[{\left(\frac{3r_{0}}{2}\right)}^{3}-{\left(\frac{r_{0}}{2}\right)}^{3}\right]$$

From this equation, *n_s_* = 26, and consequently, the fraction of bonds attributed to neighboring macromolecules within the considered cluster is determined by the value:

(13) $$\alpha =26\exp\left(-\frac{r_{0}^{2}}{{2R}_{0}^{2}}\right)$$

The maximum value of *α* corresponds to the case where the average distance between clusters equals twice the average cluster radius (concentrated solution). Computation using Formula (13) yields a value of approximately *α* ≈ 3.5 for this scenario, indicating that the concentration of “foreign” bonds within the volume corresponding to a macromolecular cluster surpasses the concentration of “self” bonds. This result may seem paradoxical at first glance, yet under Gaussian distribution, a substantial portion of the macromolecular chain extends beyond the formal boundaries set by the cluster radius. Provided that the bonds are evenly distributed in the solution volume (as is the case in a concentrated solution, where clusters are closely adjacent by the criterion of formal radii), the concentration of “self” bonds within a specific cluster can indeed be lower than the concentration of “foreign” bonds.

We shall utilize the previously formulated highly simplified model of swelling/compression of clusters, within which internal connections between chain segments are established. It is reiterated that the purpose of the proposed model is not to calculate the characteristics of hydrophobic–hydrophilic associates but to demonstrate the feasibility of their existence.

The volume of a swollen cluster containing *n* subchains, each with a length *L*, in accordance with the majority of established macromolecular cluster models (freely jointed chain, persistent chain, etc.), can be expressed as:

(14) $$V\sim nL^{3/2}$$

Here, the subchain length is related to:

(15) $$L\sim \frac{L_{0}}{n}$$

Transitioning to the number $N_{0}$ of nodes U (Figure 11) capable of forming connections with other such nodes, we obtain:

(16) $$V\sim \sqrt{\frac{N_{0}^{3}}{n}}$$

The number of subchains is linked to the concentration $q_{i}=\left[U_{2}\right]$ of interacting nodes $U_{2}$ within the cluster volume V, given as $n=2Vq_{i}$, as each additional bond leads to the division of two existing subchains. Thus,

(17) $$V=\vartheta \sqrt{\frac{N_{0}^{3}}{2q_{i}V}}$$

where *ϑ* is the proportionality coefficient. For convenience, we introduce a quantity representing the volume of a cluster devoid of subchains:

(18) $$V=\sqrt[{3}]{\frac{V_{0}^{2}}{2q_{i}}}\;V_{0}=\vartheta \sqrt{N_{0}^{3}}$$

Hence, combining Formulas (15) and (16), we derive an equation governing the relationship between the cluster volume and the concentration of bonds $q_{i}$ within it:

(19) $$V=\frac{V_{0}}{\sqrt{2q_{i}V}}$$

Notably, this equation no longer contains the proportionality coefficient *ϑ*. Solving (19) for *V* yields expressions linking the cluster volume to the bond concentration $q_{i}$:

(20) $$V=\sqrt[{3}]{\frac{V_{0}^{2}}{2q_{i}}}$$

Within the framework of the employed model, the competition between internal cluster bonding and bonding with fragments of other clusters is reflected through the following equations.

(21) $$c_{i}^{2}=Kq_{i}$$

(22) $$c_{i}c_{e}=Kq_{e}$$

(23) $$2q_{i}+q_{e}+c_{i}=c_{0}=\frac{N_{0}}{V}$$

where $c_{i}$ represents the concentration of U nodes capable of engaging in hydrophilic interactions, specifically associated with the focal cluster. $c_{e}$ denotes the concentration of such nodes, extending to the encompassing clusters. The concentration $q_{i}=\left[U_{2}\right]$ pertains to $U_{2}$ nodes of interest, correlating to those situated within a single chain, while $q_{e}$ is the analogous measure for interactions involving nodes from neighboring clusters. K symbolizes the effective equilibrium constant in the formal reaction (3), whereas $c_{0}$ encompasses the overall effective concentration of U nodes. The variable V represents the cluster’s volume.

Upon substituting the expression for the cluster’s volume (derived in Equation (20)) into Formula (23), the following relationship emerges:

(24) $$2q_{i}+q_{e}+c_{i}=c_{0}\sqrt[{3}]{2q_{i}V_{0}}$$

By subsequently substituting the expressions for the concentrations $q_{e,i}$ (21) and (22) into Equation (24), we yield:

(25) $$2c_{i}^{2}+\left(c_{e}+K\right)c_{i}=K^{2/3}c_{0}\sqrt[{3}]{2c_{i}^{2}V_{0}}$$

As previously illustrated, the connection between $c_{e}$ and $c_{i}$ can be effectively presented through the coefficient α, as given by Equation (13):

(26) $$c_{e}=\alpha c_{i}$$

By utilizing relation (22) and transitioning to the dimensionless concentration $x=\frac{c_{i}}{c_{0}}$, we obtain:

(27) $$\left(2+\alpha \right)x^{4/3}+kx^{1/3}=k^{2/3}{\left(2N_{0}\right)}^{1/3}$$

Here, $k=\frac{K}{c_{0}}$ is introduced as the dimensionless control parameter.

A correspondence between the quantity under the exponent in Equation (13) and the normalized concentration x can be established. Expression (20) can be reformulated as:

(28) $$V=\sqrt[{3}]{K\frac{V_{0}^{2}}{c_{0}^{2}x^{2}}}=\sqrt[{3}]{\frac{K}{c_{0}}\frac{N_{0}^{2}}{c_{0}^{3}x^{2}}}$$

This leads to:

(29) $$V^{-1}=c_{0}\sqrt[{3}]{\frac{c_{0}}{K}\frac{x^{2}}{N_{0}^{2}}}$$

Consequently:

(30) $${\left(\frac{r_{0}}{2R_{0}}\right)}^{2}={\left(\frac{c_{0}}{C}\sqrt[{3}]{\frac{c_{0}}{K}\frac{x^{2}}{N_{0}^{2}}}\right)}^{2/3}={\left(\frac{c_{0}}{C_{0}}\sqrt[{3}]{\frac{c_{0}}{K}N_{0}x^{2}}\right)}^{2/3}={\left(\frac{c_{0}}{C_{0}}\right)}^{2/3}{\left(\frac{c_{0}}{K}N_{0}\right)}^{2/9}x^{4/9}$$

Here, it is noted that the average bond concentration in the solution is linked to the concentration of macromolecular clusters as:

(31) $$C_{0}=CN_{0}$$

Transitioning to dimensionless variables in formula (26), we derive:

(32) $${\left(\frac{r_{0}}{2R_{0}}\right)}^{2}=\frac{\beta }{2}{\left(\frac{N_{0}}{k}\right)}^{2/9}x^{4/9}$$

Here, the central governing parameter for the contemplated model, denoted as β, is introduced:

(33) $$\beta =2{\left(\frac{c_{0}}{C_{0}}\right)}^{2/3}$$

In a physical sense, it quantifies the extent to which the average bond concentration within the cluster surpasses the overall average bond concentration across the solution’s volume. Essentially, this parameter characterizes the degree of solution heterogeneity.

Upon substituting the expression (32) into Formula (23), we arrive at the ultimate equation for the normalized concentration x:

(34) $$\left(2+26\exp\left[-\beta {\left(\frac{N_{0}}{k}\right)}^{2/9}x^{4/9}\right]\right)x^{4/3}+kx^{1/3}=k^{2/3}{\left(2N_{0}\right)}^{1/3}$$

The expression for the normalized concentration of $U_{2}$ nodes, as deduced from (13), (26), and (32), is articulated as:

(35) $$y=\frac{c_{e}}{c_{0}}=26x\exp\left[-\beta {\left(\frac{N_{0}}{k}\right)}^{2/9}x^{4/9}\right]$$

**Figure 12:**
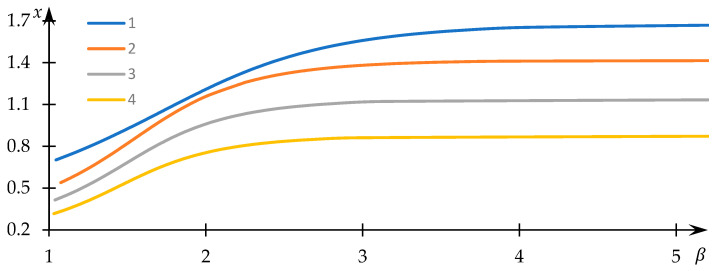
Dependency of the normalized concentration x on the control parameter β; k = 10 (1), 2 (2), 1 (3), 0.5 (1).

**Figure 13:**
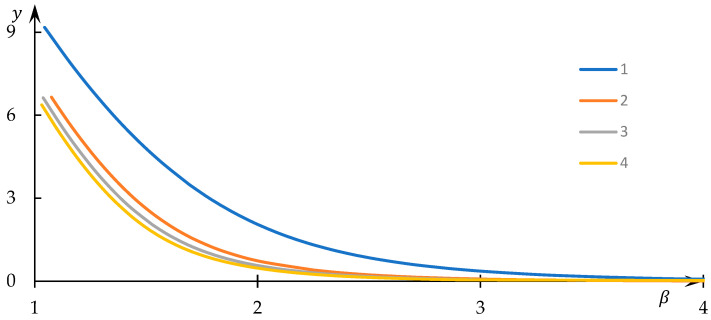
Dependency of the normalized concentration y on the control parameter β; k = 10 (1), 2 (2), 1 (3), 0.5 (1).

Examples of solutions to Equation (34) for varying values of the normalized equilibrium constant k are visually presented in Figure 12, while instances of the dependencies of the normalized concentration y on the control parameter β are graphically depicted in Figure 13. The numerical values corresponding to the obtained solutions are documented in the supporting file, where the method of substituting these numerical data into the original equation demonstrates that the obtained numerical outcomes indeed align with the solution to Equation (34).

Evidently, as the parameter β assumes larger values, the normalized concentration x tends toward a constant value, while the normalized concentration y converges to zero. This outcome aligns with expectations, given that elevated β values correspond to conditions where macromolecular clusters exhibit minimal overlap and interaction. Consequently, bonds between associated U nodes do not form, and the equilibrium of concentrations is solely governed (within the scope of the employed model) by the equilibrium in formal reaction (3).

In contrast, for comparatively smaller β values, the normalized concentration x assumes values noticeably lower than those in the aforementioned limiting case. This implies that a significant fraction of U nodes establishes interactions with equivalent nodes belonging to different macromolecules, thus leading to the formation of hydrophobic–hydrophilic associates. Moreover, Figure 12 reveals that x substantially diminishes as k decreases—an explanation readily derived: as k reduces, formal reaction (3) shifts to the right, favoring the creation of $U_{2}$ nodes. The behavior of the curves in Figure 13 with decreasing k is somewhat unexpected: it is evident that in this scenario, the normalized concentration y decreases. Nevertheless, this apparent contradiction is easily resolved, as the most efficient interaction between U nodes—belonging to distinct macromolecular clusters—occurs within sparsely populated clusters.

This deduction prompts the consideration of values of β<2. Specifically, based on the obtained results (Figure 12 and Figure 13), the region of the most effective interaction between clusters can coincide with the contact region of their peripheries. Here, the parameter β can indeed take on values β<2, forming the nucleus of the hydrophobic–hydrophilic associate. 

However, this assertion necessitates further substantiation using a more comprehensive model that accounts for the non-uniform distribution of clusters within the solution volume (it need not be demonstrated that any polymer solution constitutes a highly fluctuating system). The proposed model does not accommodate this factor.

There are substantial grounds to believe that the mentioned factor—markedly non-uniform distribution of bond concentrations within the solution volume—is most prominently manifested under conditions where the phase transition is only partial, i.e., processes stemming from changes in the hydrophobic–hydrophilic balance are inherently reversible.

**Figure 14:**
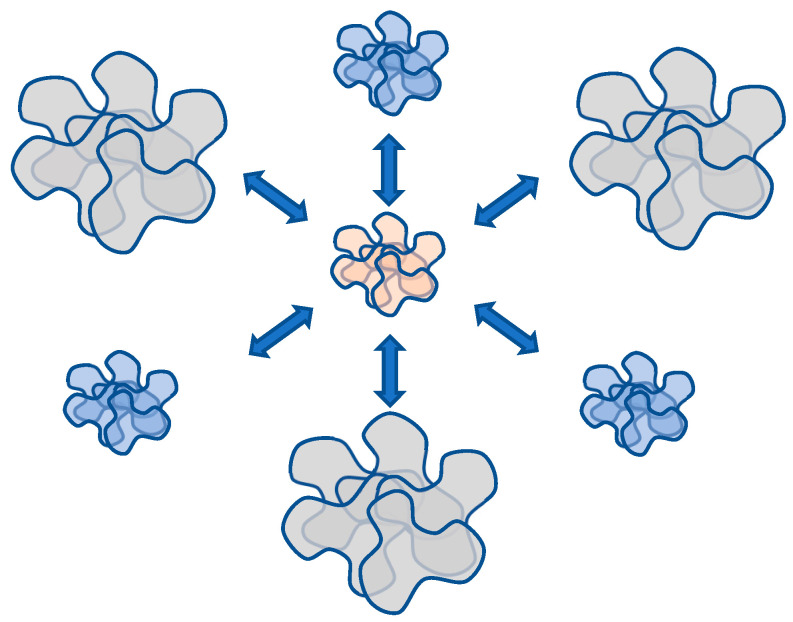
Illustration of the neural properties of solutions of the examined type.

**Figure 15:**
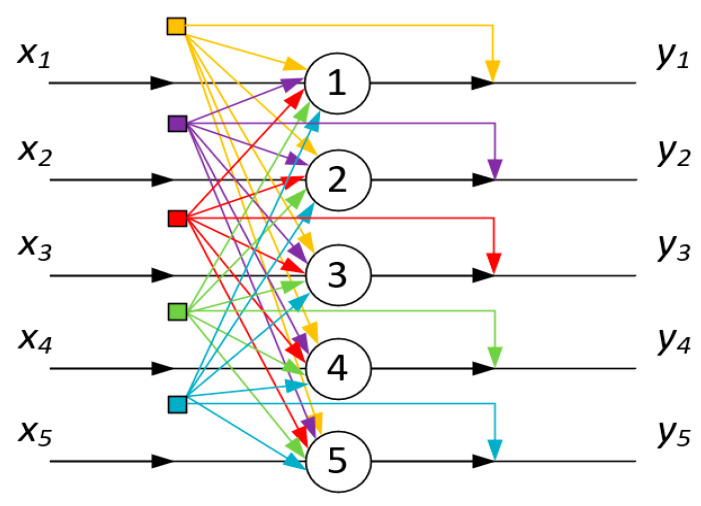
Hopfield neural processor diagram, five-neuron case, adapted from [39]; $x_{i}$—input logical variables, $y_{i}$—outputs.

Hence, a hypothesis may be posited whereby systems of the considered nature, under specific conditions, are capable of transitioning into analogues of neural networks. Indeed, in the examined conditions, a certain portion of macromolecules forms either associates or compact globules. As a result, the local concentration of unreacted nodes inevitably undergoes a change in the remaining solution volume. Figure 14 schematically illustrates this phenomenon, depicting the mutual influence of forming associates/globules on each other. In the aforementioned sense, each of the associates/globules affects all others. Therefore, in terms of topology, the scheme in Figure 14 is equivalent to the Hopfield neural processor diagram, as presented in Figure 15.

This hypothesis, in any case, with respect to the considered associates, naturally requires further confirmation. However, the presented considerations are sufficient to regard solutions of the investigated type as systems whose behavior is predominantly influenced by arising non-uniformities of a fluctuational nature.

On the whole, it is possible to propose a sufficiently straightforward model that corroborates the conclusion regarding the existence of hydrophobic–hydrophilic associates formed by macromolecules of the same type. This deduction, in turn, signifies that investigations of phase transitions of the examined nature necessitate attention to such associates that could serve as “nuclei” for the formation of insoluble products. Furthermore, the derived inference signifies the existence of circumstances in which fluctuations or heterogeneities within the polymer solution substantially impact its behavior, even to the extent of manifesting properties akin to neural networks.

## 5. Conclusions

Hence, our proposed approach to analyzing turbidimetric measurements facilitates the discernment of hydrophobic–hydrophilic associations, arising as a result of amphiphilic interactions among macromolecules within the phase transition region. The presence of such associations is further validated through an illustrative model introduced in this study.

The configuration of these associations bears notable resemblance to micellar structures formed by surfactant molecules within aqueous solutions. These associations encompass a relatively compact hydrophobic core stabilized by hydrophobic forces, enabling the scattering of optical radiation. Surrounding this core is a hydrophilic periphery that averts the precipitation of the association.

The genesis of hydrophobic–hydrophilic associations under the examined conditions can be attributed to forces instigating the expansion of macromolecular coils (osmotic pressure). If the influence of hydrophobic interactions is inadequate to counterbalance these forces, coil compaction is impeded. Consequently, favorable energetic interactions manifest between distinct macromolecules, culminating in the formation of associations of the aforementioned type. Notably, their salient attribute is their responsiveness to components present in the solution capable of modifying the hydrophobic–hydrophilic equilibrium coupled with their capacity for optical radiation scattering.

## Data Availability

The authors confirm that the data supporting the findings of this study are available within its supplementary materials.

## Supplementary Materials

The following supporting information can be downloaded at: https://www.mdpi.com/article/10.3390/polym15173578/s1.
